# Chronic opioid use following surgery for head and neck cancer patients undergoing free flap reconstruction

**DOI:** 10.1186/s40463-021-00508-y

**Published:** 2021-04-23

**Authors:** Ashley Hinther, Alysha Rasool, Steven C. Nakoneshny, Shamir P. Chandarana, Robert Hart, T. Wayne Matthews, Joseph C. Dort

**Affiliations:** 1 Department of Surgery, Section of Otolaryngology- Head and Neck Surgery, Cumming School of Medicine, University of Calgary, HRIC 2A02 3280 Hospital Dr NW, Calgary, Alberta T2N 4Z6 Canada; 2 Ohlson Research Initiative, Arnie Charbonneau Cancer Institute, Cumming School of Medicine, University of Calgary, Calgary, Alberta Canada

**Keywords:** Head and neck surgery, Postoperative pain, Opioids, Postoperative opioid use

## Abstract

**Background:**

Physician opioid-prescribing patterns have significant impacts on the current opioid crisis. Patients who use opioids in the postoperative period are at risk of developing chronic postoperative opioid use. This study determined the rate of chronic postoperative opioid use among head and neck cancer patients undergoing primary surgery with free-flap reconstruction. Additionally, this study identified major risk factors associated with the development of chronic postoperative opioid use.

**Methods:**

A retrospective chart review was performed for all adults (age ≥ 18 years) undergoing primary head and neck surgical resection with free-flap reconstruction between January 2008 and December 2015. Patients were identified from a prospectively collected database, Otobase™. Data from the provincial drug insurance program were used to capture drug dispensing information to determine chronic opioid use at 3- and 12-months postoperatively. Data extracted from Otobase™ included patient demographics, social habits, clinical stage, pathological stage, type of surgery, and adjuvant treatment.

**Results:**

The total cohort was comprised of 212 patients. Chronic opioid use at 3- and 12- months postoperatively was observed in 136 (64%) and 116 (55%) patients, respectively. Of the 212 patients, 85 patients (40%) were identified as preoperative opioid users and 127 were opioid naïve (60%). Of the 85 patients who were preoperative opioid users, 70 (82%) and 63 (77%) patients continued to use opioids 3- and 12-months postoperatively, respectively. The proportion of opioid-naïve patients who were using opioids at 3- and 12-months postoperatively was 52% (66 patients) and 42% (53 patients), respectively. Identified risk factors included preoperative opioid use, prior tobacco use, advanced pathologic T-stage, and adjuvant treatment.

**Conclusions:**

Among head and neck cancer patients that have undergone major resection with free-flap reconstruction, the prevalence of chronic postoperative opioid users was considerable. Identified risk factors included preoperative opioid use, prior tobacco use, tumor stage, and adjuvant treatment.

**Graphical abstract:**

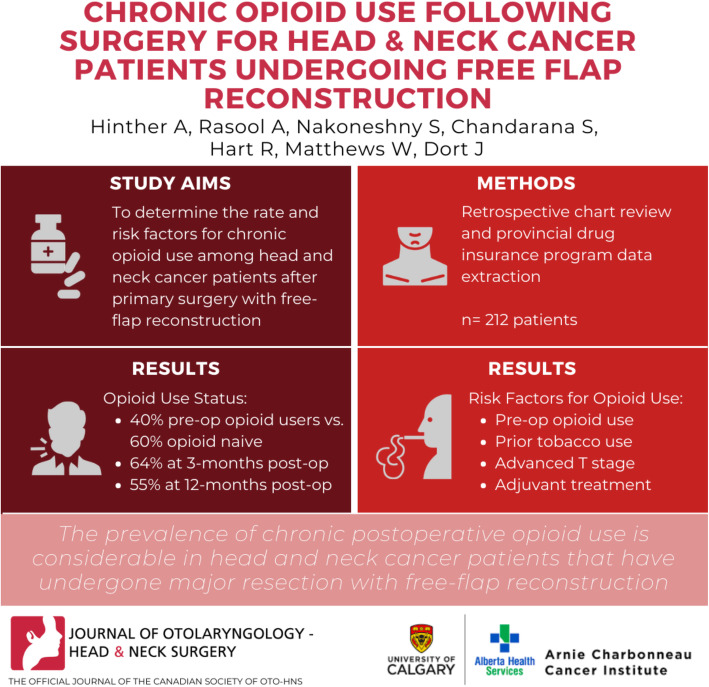

## Background

An opioid-associated public health emergency exists, in part, because of a dramatic rise in opioid-prescriptions and opioid-related deaths [1]. Within the past year, the number of deaths in Canada from accidental overdose secondary to opioids has increased by greater than 40%. Opioids are now the most frequently prescribed drug class in the United States [2, 3]. Deaths from prescription opioids in the United States quadrupled between 2000 and 2014, and are correlated with a concurrent 4-fold increase in the quantity of opioids dispensed [3]. Most opioid abusers receive opioids through a diversion of an appropriately-obtained drug; 1 in 4 cancer patients admit to losing or sharing prescribed opioids [1, 4, 5]. Furthermore, there is a direct relationship between the number of opioid overdoses within a community and the quantity of prescription drugs dispensed [6].

The origin of the opioid epidemic is partly secondary to an effort put forth by the American Pain Society in the 1990s for better in-hospital pain management [7, 8]. The labeling of pain as a “fifth vital sign” resulted in the marketing of opioids as a superior alternative to other, non-addictive analgesics. Opioid medications provide an effective means for managing acute postoperative pain; however, opioids are associated with significant side-effects including respiratory depression, constipation, nausea and vomiting, and impaired mobilization [9, 10]. Postoperative opioid use has also been linked to the subsequent development of opioid dependency; therefore, the postoperative period represents a challenging situation in optimizing pain control while minimizing the risks of chronic opioid use [11, 12].

Chronic postoperative opioid use is defined as continued opioid consumption greater than 3-months after surgery [13]. The causes of postoperative chronic opioid use are complex and multifactorial. The magnitude of surgery may not necessarily be the strongest predictive factor for the development of chronic postoperative opioid use [13, 14]. Opioid dependency following orthopedic procedures is well described in the literature, yet little is known about opioid dependency following major head and neck reconstructive surgery [15–18].

Major head and neck cancer resections with free flap reconstruction are lengthy and complex procedures and patients often require nasogastric and tracheotomy tubes. These interventions have a major impact on postoperative patient comfort and can make pain management challenging. In most head and neck centers, narcotic analgesics form a major component of postoperative pain control regimens [19, 20]. In our center, similar to others, most patients are managed with intravenous patient-controlled analgesia (PCA) for the first five postoperative days and subsequently switched to a combination of narcotic and non-narcotic analgesics. Pain management following major head and neck surgery therefore represents a complex challenge, wherein opioids have traditionally been a mainstay in management [12]. Little is known about the prevalence of chronic opioid use in this patient population. The aim of this study, therefore, is to determine the prevalence of, and risk factors contributing to, chronic postoperative opioid use following head and neck surgery with free-flap reconstruction.

## Methods

### Patient selection

**Fig. 1:**
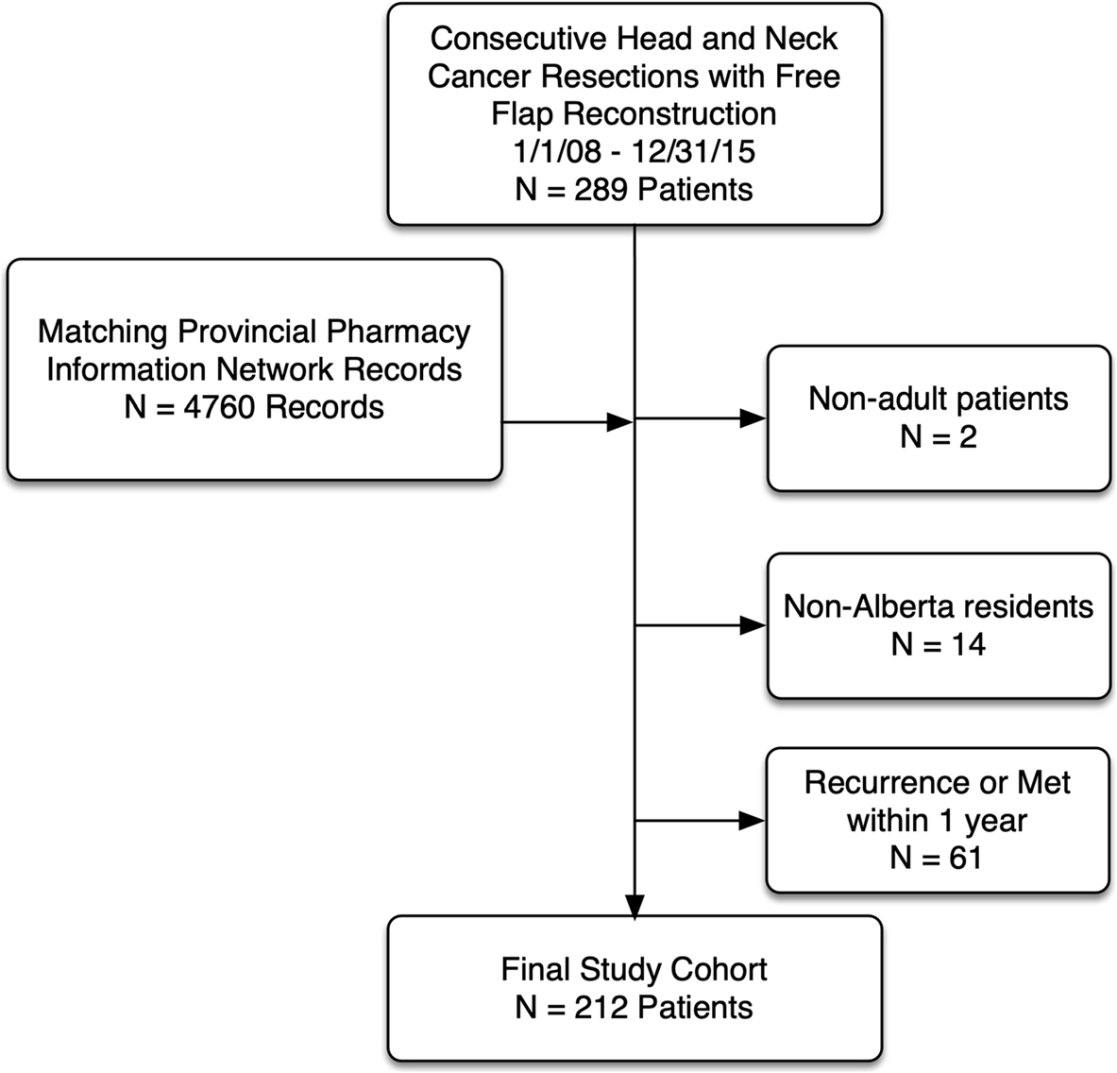
Study CONSORT diagram

We performed a retrospective chart review at a tertiary, academic head and neck surgical oncology program (University of Calgary, Calgary, Alberta, Canada), on all adults (age ≥ 18 years) undergoing primary head and neck surgical resection with free-flap reconstruction from January 2008 – December 2015. Eligible patients met the following criteria: age ≥ 18 years old undergoing primary surgical resection with free-flap reconstruction, residents of Alberta, Canada, and patients who survived greater than 1 year following hospital discharge, no recurrence within 1 year of surgery, a second primary malignancy or documented metastases within the first postoperative year. Exclusion criteria included: patients undergoing surgery for recurrence, age < 18 years old, and patients who were not Alberta residents. Non-Alberta residents were excluded as their pharmacy information was not readily available. Figure 1 is a Consolidated Standards of Reporting Trials (CONSORT) diagram describing the cohort.

### Data collection

Data extracted from Otobase™ included patient demographics, prior tobacco and alcohol use (dichotomized into never/ever), clinical stage, pathological stage, surgical details, and adjuvant treatment. We collected medication data from the Provincial Pharmacy Information Network (PPIN) that included the following information: drugs prescribed, date dispensed, quantity supplied, unit of measure, days supplied per script, DIN, oral morphine equivalents (OME (mg)), and prescriber information. Preoperative opioid use was defined as those who had filled at least one opioid prescription between 31 days and 12-months preoperatively. All drugs classified as opioids were included in the analysis (in descending order of prevalence: codeine, hydromorphone, morphine, oxycodone, fentanyl, tramadol, buprenophrine, meperidine, and tapentadol). Chronic opioid use was defined as filling an opioid prescription more than 90 days after surgery [13]. Data was also collected on patients receiving ongoing opioid prescriptions greater than 12-months postoperatively. The primary outcomes of the study included the prevalence of opioid use at 3- and 12-months postoperatively. Secondary outcomes of the study were factors associated with chronic postoperative opioid use through univariate and multivariate analysis, and the number of prescribing physicians.

### Statistical analysis

Categorical data were analyzed using a chi-square or Fisher’s exact test, as appropriate. Continuous data were compared using appropriate parametric or nonparametric analyses. Variables of interest for regression modelling were included in a series of univariate probes. Univariate analyses achieving *p*-values ≤0.2 were included in the multivariate models. All statistical analyses were performed using Stata 16.0 (StataCorp LP, College Station, Tx). Two-sided tests were used to analyze all data. A *p*-value of ≤0.05 was considered significant.

### Ethical approval

The authors used A Project Ethics Community Consensus Initiative (ARECCI) framework to assess for and mitigate ethical risks, including the ARECCI Ethics Screening Tool and the ARECCI Ethics Guidelines. The project was deemed a quality improvement initiative with a minimal risk (ARECCI score = 2) [21].

## Results

The clinical characteristics of the cohort (*n* = 212) are shown in Table 1. The mean age overall was 61 years with a range of 19.5–89.8 years. Prior alcohol use was observed in 145 (68%) patients, and prior tobacco use was observed in 148 (70%) patients. Eighty-five (40%) patients were classified as preoperative opioid users. The majority of patients presented with Stage IV disease (108 patients (51%)). The most common primary site was the oral cavity (164 patients (77%)). One hundred and ninety-eight (93%) patients received either a soft tissue or osseous free-flap and 14 patients (7%) were reconstructed with both a soft tissue and osseous free-flap. All patients underwent primary surgical resection and 120 (57%) were treated with adjuvant therapy.

**Table 1:** Patient demographics and clinical characteristics

Clinical Characteristic	Patients *N* = 212 (%)		
	Preoperative Opioid-Naïve	Preoperative Opioid Use	***p*** value
Total	*n* = 127 (60%)	*n* = 85 (40%)	
Gender			0.046
Male	96 (76%)	53 (62%)	
Female	31 (24%)	32 (38%)	
Age (years)			0.8
Mean [SD]	61.2 [13.81]	60.6 [12.66]	
Range	19.5–89.8	23.4–86.6	
Social Habits			
Prior Tobacco	89 (70%)	59 (69%)	1.0
Prior Alcohol	87 (69%)	58 (68%)	0.8
Clinical Stage			0.05
In Situ (Stage 0)	2 (2%)	1 (1%)	
I	11 (9%)	15 (18%)	
II	26 (20%)	13 (15%)	
III	15 (12%)	13 (15%)	
IV	65 (51%)	43 (51%)	
Other	8 (6%)	0 (0%)	
Primary Tumour Location			0.04
Oral Cavity	102 (80%)	62 (73%)	
Salivary Gland	4 (3%)	8 (9%)	
Paranasal Sinus	4 (3%)	8 (9%)	
Other	17 (13%)	7 (8%)	
Free Flap			1.0
Soft Tissue	95 (75%)	64 (75%)	
Bone	23 (18%)	16 (19%)	
Soft Tissue + bone	9 (7%)	5 (6%)	
Treatment			0.6
Surgery Alone	53 (42%)	39 (46%)	
Surgery + Adjuvant	74 (58%)	46 (54%)	

Chronic opioid use at 3- and 12-months postoperatively was observed in 136 (64%) and 116 (55%) patients, respectively (Table 2). Eighty-five patients (40%) used opioids preoperatively and, of those, 70 (82%) and 63 (74%) patients continued to use opioids at 3- and 12-months postoperatively, respectively. Pre-operative opioid use was significantly related to 3- and 12-month post-operative opioid use (*p* < 0.0001).

**Table 2:** Proportion of patients with chronic postoperative opioid use

	**3 m + Chronic Postoperative Opioid Use**			
	Yes	No	Total	***p*** **value**
Naïve	66	61	127	
Non-naïve	70	15	85	
Total	136	76	212	***p*** **< 0.0001**
	**12 m + Chronic Postoperative Opioid Use**			
	Yes	No	Total	***p*** **value**
Naïve	53	74	127	
Non-naïve	63	22	85	
Total	116	96	212	***p*** **< 0.0001**

Table 3 describes the clinical characteristics of chronic opioid users at 3- and 12-months postoperatively. There was a higher proportion of oral cancer in the 3-month chronic opioid use group (*p* = 0.03).

**Table 3:** Clinical characteristics of patients using chronic postoperative opioids

Clinical Characteristic	3 m + Chronic Postoperative Opioid Use (***N*** = 136)			12 m + Chronic Postoperative Opioid Use (***N*** = 116)		
	Naïve *N* = 66 (%)	Non-Naïve *N* = 70 (%)	***p*** value	Naïve *N* = 53 (%)	Non-Naïve *N* = 63 (%)	***p*** value
**Gender**			0.3			0.7
Male	44 (63%)	47 (71%)		38 (72%)	42 (67%)	
Female	26 (37%)	19 (29%)		15 (28%)	21 (33%)	
**Age (years)**			0.7			0.6
Mean [SD]	62.3 [12.34]	61.6 [10.79]		62.7 [12.23]	61.6 [10.93]	
Range	31.4–88.2	23.4–82.0		31.8–88.2	23.4–82.0	
**Social Habits**						
Prior Tobacco	53 (80%)	53 (76%)	0.6	43 (81%)	46 (73%)	0.4
Prior Alcohol	49 (74%)	49 (70%)	0.3	39 (74%)	43 (68%)	0.5
**Clinical Stage**			0.08			0.2
In Situ (Stage 0)	0 (0%)	1 (1%)		0 (0%)	1 (2%)	
I	7 (11%)	13 (19%)		6 (11%)	13 (21%)	
II	14 (21%)	8 (11%)		12 (25%)	8 (13%)	
III	4 (6%)	11 (16%)		3 (6%)	7 (11%)	
IV	41 (62%)	37 (53%)		31 (59%)	34 (54%)	
**Primary Tumour Location**			**0.03**			0.06
Oral Cavity	59 (89%)	48 (69%)		47 (89%)	43 (68%)	
Salivary Gland	2 (3%)	7 (10%)		1 (2%)	7 (11%)	
Paranasal Sinus	2 (3%)	8 (11%)		2 (4%)	7 (11%)	
Other	3 (5%)	7 (10%)		3 (6%)	6 (10%)	
**Free Flap**			1			0.9
Soft Tissue	49 (74%)	52 (74%)		41 (77%)	47 (75%)	
Bone	13 (20%)	14 (20%)		10 (19%)	13 (21%)	
Soft Tissue + bone	4 (6%)	4 (6%)		2 (4%)	3 (5%)	
**Treatment**			0.2			0.4
Surgery Alone	19 (29%)	29 (41%)		19 (36%)	28 (44%)	
Surgery + Adjuvant	47 (71%)	41 (59%)		34 (64%)	35 (56%)	

In univariate logistic regression (Table 4), patients younger than 40 years of age were less likely to continue using opioids at 3- and 12-months postoperatively (OR 0.25, 95% CI [0.09–0.69), *p* < 0.007; OR 0.29, 95% CI [0.10–0.84], *p* = 0.02, respectively). Tobacco users were more likely to demonstrate chronic opioid use at 3-months and 12-months postoperatively versus non-tobacco users (OR 2.52, 95% CI [1.34–4.76], *p* = 0.004; OR 1.87 95% CI [1.01–3.48] *p* = 0.048, respectively). Advanced pathologic T-stage was significantly associated with 3-month postoperative chronic opioid use (OR 2.27, 95% CI [1.24–4.15], *p* = 0.008). Patients undergoing both surgery and adjuvant therapy were nearly three times as likely to continue using opioids 3-months postoperatively (OR 2.52, 95% CI [1.42–4.48] *p* < 0.002) compared to patients undergoing surgery alone. Preoperative opioid use was the strongest predictor of postoperative use at both 3- and 12-months postoperatively (OR 4.31, 95% CI [2.24–8.32], *p* < 0.0001; OR 4.00, 95% CI [2.19–7.29], *p* = 0.0001, respectively).

**Table 4:** Univariate and Multivariate logistic regression analysis of factors associated with chronic postoperative opioid use

Clinical Characteristic	3 m + Persistent Opioid Use		12 m + Persistent Opioid Use	
	Odds Ratio [95% CI]	***p***-value	Odds Ratio [95% CI]	***p***-value
**Univariate Probes**				
**Gender (Ref: Female)**	1.59 [0.84–3.02]	0.152	1.15 [0.64–2.08]	0.6
**Age (years) (centered at mean)**	1.01 [0.99–1.04]	0.153	1.01 [0.99–1.04]	0.168
≥ 65	1.04 [0.59–1.85]	0.9	1.04 [0.60–1.81]	0.9
55–64	1.34 [0.72–2.50]	0.4	1.44 [0.79–2.61]	0.2
40–54	1.31 [0.65–2.65]	0.5	1.04 [0.54–2.02]	0.9
< 40	0.25 [0.09–0.69]	**0.007**	0.29 [0.10–0.84]	**0.022**
**Pre-operative opioid use**	4.31 [2.24–8.32]	**< 0.0001**	4.00 [2.19–7.29]	**< 0.0001**
**Social Habits**				
Prior Tobacco	2.52 [1.34–4.76]	**0.004**	1.87 [1.01–3.48]	**0.048**
Prior Alcohol	1.24 [0.61–2.51]	0.6	1.13 [0.57–2.24]	0.7
**Advanced pathologic T-stage (T3/4)**	2.27 [1.24–4.15]	**0.008**	1.21 [0.70–2.12]	0.5
**Primary Tumour Location (ref: Oral Cavity)**				
Salivary Gland	1.60 [0.42–6.14]	0.5	1.64 [0.48–5.68]	0.4
Paranasal Sinus	2.66 [0.56–12.57]	0.2	2.47 [0.64–9.44]	0.187
Other	0.38 [0.16–0.91]	**0.03**	0.49 [0.20–1.19]	0.1
**Free Flap (Ref: Soft Tissue)**				
Bone	1.29 [0.61–2.74]	0.5	1.15 [0.57–2.36]	0.7
Soft Tissue + Bone	0.77 [0.25–2.32]	0.6	0.45 [0.14–1.40]	0.167
**Treatment (ref: Sx Alone)**				
Sx + Adjuvant	2.52 [1.42–4.48]	**0.002**	1.30 [0.75–2.24]	0.4
**Multivariate Model**				
**Gender (Ref: Female)**	2.15 [0.92–5.01]	0.08	–	–
**Age (years) (centered at mean)**	0.99 [0.96–1.03]	0.7	1.00 [0.97–1.03]	0.8
< 40	0.25 [0.04–1.35]	0.1	0.37 [0.08–1.76]	0.2
**Pre-operative opioid use**	4.02 [1.85–8.72]	**< 0.0001**	2.34 [1.15–4.74]	**< 0.0001**
**Social Habits**				
Prior Tobacco	2.80 [1.23–6.38]	**0.014**	2.34 [1.15–4.74]	**0.019**
**Advanced pathologic T-stage (T3/4)**	1.70 [0.79–3.69]	0.2	–	–
**Primary Tumour Location (ref: Oral Cavity)**				
Salivary Gland	0.63 [0.14–2.95]	0.6	1.09 [0.28–4.35]	0.9
Paranasal Sinus	1.91 [0.27–13.42]	0.5	1.99 [0.42–9.36]	0.4
Other	0.37 [0.12–1.15]	0.09	0.44 [0.15–1.28]	0.1
**Free Flap (Ref: Soft Tissue)**				
Bone	–	–	1.25 [0.56–2.80]	0.6
Soft Tissue + Bone	–	–	0.33 [0.09–1.19]	0.09
**Treatment (ref: Sx Alone)**				
Sx + Adjuvant	2.23 [1.03–4.82]	**0.042**	–	–

Multivariable logistic regression (Table 4) revealed that preoperative opioid use was associated with postoperative opioid use at both 3- and 12-months postoperatively (OR 4.02, 95% CI [1.85–8.72], p < 0.0001; OR 2.34, 95% CI [1.15–4.74], p < 0.0001, respectively). A history of tobacco use was also independently associated with chronic postoperative opioid use at 3-months and 12-months after surgery (OR 2.80, 95% CI [1.23–6.38], *p* = 0.014; OR 2.34 95% CI [1.15–4.74], *p* = 0.019). Treatment with surgery and adjuvant therapy was independently associated with chronic postoperative opioid use at 3-months only (OR 2.23, 95% CI [1.03–4.82], *p* = 0.042).

Patients who were chronic opioid users at 12-months postoperatively had a significantly higher number of physicians prescribing opioids compared to patients who were not chronic opioid users. In chronic users, the mean number of prescribers at 3- and 12 months postoperative was 2.6 (*p* = 0.08) and 2.8 (*p* < 0.0027), respectively. The mean number of prescribers for patients who were no longer using opioids at 3- and 12-months was 1.7 for both time points.

## Discussion

This study examined the prevalence of chronic opioid use following primary head and neck cancer surgery with free-flap reconstruction. We found a high proportion of patients using opioids at 3- and 12-months postoperatively (67 and 48%, respectively). In an unadjusted analysis, chronic postoperative opioid use was strongly associated with preoperative opioid use, a history of tobacco use, advanced pathologic T-stage, and postoperative adjuvant therapy. Multivariate analysis revealed that preoperative opioid use and multimodality treatment were significantly associated with chronic postoperative opioid use.

A recent retrospective study by Smith et al. investigated prolonged opioid use (6 months after treatment completion) in head and neck patients undergoing curative-intent radiation therapy (RT). These authors found that RT given in an adjuvant setting was associated with a lower probability of opioid use at 6 months after completion of RT. Our study found that adjuvant therapy was associated with increased opioid use at 3 months but this association was no longer seen at 12 months after treatment [22].

There is limited research evaluating the prevalence of chronic postoperative opioid use in the head and neck cancer population. Pang et al. studied the prevalence of chronic postoperative opioid use at 3-months following primary surgical resection for oral cavity cancer and 41% of patients continued to use opioids at 3-months postoperatively with 82% of the patients citing head and neck cancer pain as the primary reason for continued opioid use [18]. Our study results are slightly higher than previously reported results, likely secondary to our cohort’s clinical characteristics. A high proportion of our patient population (47%) had a pT-stage of T3 or higher, versus 34% of patients reported by Pang et al. Furthermore, our study demonstrated advanced pathologic T-stage to be significantly associated with chronic postoperative opioid use at 3 months postoperatively.

Our study demonstrated preoperative opioid use to be the strongest predictor of opioid use at 3 and 12-months postoperatively, consistent with previously reported results across all surgical specialties [13]. Multiple studies have demonstrated poor pain control in the head and neck cancer population despite heavy utilization of opioids. This suggests that opioid use alone is an ineffective strategy in head and neck cancer postoperative pain management [13, 18, 23]. However, chronic postoperative opioid use is complex and the risk of developing postoperative chronic opioid use is not solely related to the degree of postoperative pain. Brummett et al. compared the incidence of new chronic opioid use in patients undergoing major versus minor surgical procedures and found the incidence of chronic postoperative opioid use did not differ between major and minor surgical procedures [14]. Mudumbaje et al. studied a variety of surgical procedures and reported that prior opioid use was the best predictor of postoperative opioid use, independent of surgical procedure [24].

Our study also found that tobacco and adjuvant treatment were risk factors for chronic postoperative opioid use, which is consistent with previous reports [13]. We also found that patients younger than age 40 were less likely to become chronic opioid users. This finding conflicts with studies that show younger age is more commonly associated with chronic postoperative opioid use [13]. However, the definition of younger versus older age is inconsistent in the literature. Several factors unrelated to pain, such as physicians’ prescribing patterns, may also play a role in the development of chronic postoperative opioid use in our cohort. Patients who continued to use opioids at 3- and 12-months postoperatively had a significantly higher number of prescribers compared to patients who were no longer using opioids. This is an interesting finding that suggests patients using opioids chronically may be seeking treatment from multiple providers.

Despite the frequent use of opioid-based analgesics, pain management is ineffective in over 50% of cancer patients and the highest pain prevalence is found in patients with head and neck cancer (70%) [25]. Our centre recently evaluated the effectiveness of postoperative pain management in a head and neck cancer surgery population. We found that 85% of our patients, managed primarily with opioids, experienced ineffective pain control during some portion of their hospital stay [12]. These data suggest that current approaches to postoperative pain management in head and neck surgery are not optimal and the risks of opioid use may outweigh the potential benefits.

Multimodal analgesia is an effective method of controlling postoperative pain and can help reduce the use of opioids and subsequent adverse events such as the development of chronic postoperative opioid use [26, 27]. Recently Eggerstedt et al. demonstrated that multimodal analgesia significantly improved postoperative pain compared to narcotics alone in head and neck cancer surgery with free flap reconstruction, while also demonstrating a significant reduction in narcotic consumption in the immediate postoperative period and at discharge [28]. Appropriate preoperative counselling of patients to manage expectations pertaining to the expected postoperative pain experience is also an essential component of effective postoperative pain management.

Our study confirms the finding, as in other surgical populations, that preoperative opioid use strongly predicts chronic postoperative opioid use [13]. A novel finding arising from our study is the association between multimodality head and neck cancer treatment and chronic postoperative opioid use. Adjuvant therapy was only associated with opioid use at 3-months and not 12-months and therefore we believe the adjuvant treatment had an impact on pain. While patients are undergoing adjuvant treatment it is important to assess and treat their pain effectively, while also minimizing the risk of chronic opioid use. We believe this information will help clinicians identify patients at higher risk of chronic postoperative opioid use and perhaps implement strategies that will reduce the risk of ongoing opioid use. At our regional cancer centre, the data from this study has helped to triage those high-risk patients towards earlier visits with our acute and chronic pain service, so as to mitigate chronic opioid use at an earlier stage in the patients’ treatment.

The present study neither explored reasons for persistent opioid use nor the amount of opioids consumed before or after surgery. This may be an important question that could be addressed through future research but was not the primary focus of the current work. Our study is also limited by its analysis of opioid-dispensing patterns only, with the assumption that this corresponds to consumption patterns; in some instances, this may not be true. However, patients filling an opioid prescription are most likely using the prescribed drug. Finally, we did not adjust the followup time for patients who received adjuvant therapy because our data show that adjuvant and non-adjuvant patients were not different in their opoid consumption at 12 months. This study is strengthened by its use of prospectively collected clinical data and the comprehensive Provincial Pharmacy Information Network (PPIN) used to capture drug-dispensing information. Such data are highly reliable, and we are confident in its accuracy and reliability.

## Conclusions

In conclusion, patients undergoing major head and neck surgery are at risk for developing chronic postoperative opioid use. Our study identified several risk factors that are associated with chronic opioid use, primarily preoperative opioid use and multimodality treatment. The underlying mechanism for chronic postoperative opioid use is likely multifactorial. With this information we believe clinicians will become more aware of patients at a potentially higher risk of continued opioid use postoperatively and consider implementing strategies to help reduce peri-operative and chronic postoperative opioid use.

## Data Availability

The datasets used and/or analysed during the current study are available from the corresponding author on reasonable request.

## Abbreviations

PPIN: Provincial Pharmacy Information Network
OME: Oral Morphine Equivalents
ARECCI: A Project Ethics Community Consensus Initiative
CONSORT: Consolidated Standards of Reporting Trials
